# Novel histone post-translational modifications in Alzheimer’s disease: current advances and implications

**DOI:** 10.1186/s13148-024-01650-w

**Published:** 2024-03-09

**Authors:** Yuanyuan Qin, Ping Yang, Wanhong He, Dongze Li, Lisha Zeng, Junle Li, Tingting Zhou, Juan Peng, Ling Cao, Wei Huang

**Affiliations:** 1 Department of Nephrology, The Affiliated Hospital of Southwest Medical University, 25 Taiping Rd, Jiangyang District, Luzhou, 646000 Sichuan People’s Republic of China; 2 Department of Endocrinology and Metabolism, The Affiliated Hospital of Southwest Medical University, 25 Taiping Rd, Jiangyang District, Luzhou, 646000 Sichuan People’s Republic of China; 3 Metabolic Vascular Disease Key Laboratory of Sichuan Province, The Affiliated Hospital of Southwest Medical University, Luzhou, 646000 Sichuan China; 4 Sichuan Clinical Research Center for Diabetes and Metabolic Diseases, Luzhou, 646000 Sichuan China; 5 Sichuan Clinical Research Center for Nephropathy, Luzhou, 646000 Sichuan China; 6 Department of Rehabilitation, The Affiliated Hospital of Southwest Medical University, Luzhou, 646000 Sichuan China

**Keywords:** Alzheimer’s disease, Novel histone post-translational modifications, Histone

## Abstract

Alzheimer’s disease (AD) has a complex pathogenesis, and multiple studies have indicated that histone post-translational modifications, especially acetylation, play a significant role in it. With the development of mass spectrometry and proteomics, an increasing number of novel HPTMs, including lactoylation, crotonylation, *β*-hydroxybutyrylation, 2-hydroxyisobutyrylation, succinylation, and malonylation, have been identified. These novel HPTMs closely link substance metabolism to gene regulation, and an increasing number of relevant studies on the relationship between novel HPTMs and AD have become available. This review summarizes the current advances and implications of novel HPTMs in AD, providing insight into the deeper pathogenesis of AD and the development of novel drugs.

## Highlights

- Histone post-translational modifications (HPTMs) have been shown to be involved in the pathological mechanism of Alzheimer’s disease (AD) in a variety of models.
- The relationship between novel HPTMs and diseases has become a research hotspot.
- Studies hinted that novel HPTMs are involved in the pathogenesis of AD.

## Introduction

Alzheimer’s disease (AD), a degenerative disease of the central nervous system, is characterized by progressive cognitive and behavioral impairment, and it primarily occurs in the elderly and pre-elderly [1]. The incidence, prevalence, mortality, and morbidity rates of AD are not optimistic [2]. Multiple-related mechanisms contribute to the pathogenesis of AD, including interconnected networks of genetic, epigenetic, biological, and environment factors [3]. Deposition of misfolded amyloid beta (Aβ) peptides and the microtubule-associated protein tau are important pathological features of AD [1, 4]. Neuroinflammation and the activation and action of innate immune cells are also involved in the pathophysiological mechanisms of AD [5]. Aβ and tau are the most promising prospective drug targets for AD treatment [6]. Currently, drugs approved for AD treatment mainly provide symptomatic treatment, and their effects are often unsatisfactory [6]. The U.S. food and drug administration (FDA) has approved early-stage drugs for AD, such as anticholinesterase inhibitors and N-methyl-D-aspartate receptor antagonists, which provide only short-term symptom improvement without preventing disease progression [7]. In recent years, with the expansion of research field, amyloid-related therapy has emerged as an important trend in the future clinical trials of new drugs [8]. The FDA has approved Aducanumab and Lecanemab, which are antibodies against amyloids that may prevent or reverse the progression of AD [8]. However, this novel amyloid-related therapy has limitations because of its treatment management mode, costly monitoring, and the need for professional equipment and imagery scanning [8]. It is thus particularly urgent to explore the deeper pathogenesis of AD and develop new treatments that can prevent or slow disease progression.

Epigenetics is the bridge between the environment and heredity. Environmental changes often lead to epigenetic changes, eventually leading to disordered cellular gene expression and disease. Histone modification is a significant component of epigenetics, changing chromosome structure through acetylation, phosphorylation, methylation, and other modifications, thus affecting gene transcription and expression [9]. The advent of ultrasensitive mass spectrometry and the development of protein-modifying antibodies have facilitated the successive discovery of multiple novel histone lysine modifications, including crotonylation (Kcr) [10], lactoylation (Kla) [11], β-hydroxybutyrylation (Kbhb) [12], succinylation (Ksucc) [13], 2-hydroxyisobutyrylation (Khib) [14], and malonylation (Kmal) [15]. Over the last decade, numerous studies have explored the relationship between AD and these novel HPTMs. This review briefly summarizes the latest advances in novel HPTMs in AD, providing a fresh perspective on the development of novel targeted drugs.

## Overview of previous research on HPTMs and AD

As shown in Table 1, many previous studies have investigated HPTMs and their correlation with AD, including acetylation (Kac), methylation (Kme), phosphorylation (P), and ubiquitination (Kub).

**Table 1:** Summary of classical HPTMs in AD

HPTMs	Models	Sites	Specific functions	References
Acetylation	Human brain	H3K9ac	H3K9ac is related to tau-related pathology and chromatin remodeling	[81]
	Human brain	H3K9ac, H3K27ac, H3K122ac	1. H3K9ac and H3K27ac increase and H3K122ac reduction in AD 2. H3K9ac and H3K27ac are related to transcription, chromatin, and disease pathways	[20]
	Human brain	H3K27ac	H3K27ac is associated with transcriptional variation at proximal genes	[19]
	Human brain	H4K16ac	H4K16ac reduction in AD	[21]
	Mice	H4K5ac, H4K12ac	App specifically regulates H4K5ac and H4K12ac and affects early memory-related genes in memory	[82]
	Mice	H4 acetylation	4-PBA treatment enhances the expression of genes related to induced learning and memory genes by increasing neuronal H4 acetylation	[83]
	Human brain	H3 acetylation, H4 acetylation	H3 acetylation and H4 acetylation increase in high-pathological areas, with no significant change observed in low-pathological areas	[84]
	Mice	H4 acetylation	APP/PS1 mice display a reduced H4 acetylation levels in response to a learning task	[85]
	Mice	H3K9ac, H4K12ac	ACSS2 downregulation mediates a reduction in glutamate receptor expression through histone acetylation, which exacerbates synaptic plasticity impairment in AD	[22]
	IPSC cell lines	H3K27ac	Knocking down P300/CBP reduces H3K27ac, inhibits the expression of genetic programs compensating for increased Aβ load, and leads to increased Aβ secretion	[23]
	Drosophila	H4 acetylation	H4 acetylation may act as a defense against AD pathology-related insults	[25, 26]
Methylation	Human brain, Mice	H3K9me2	Repressive H3K9me2 and euchromatic histone methyltransferases EHMT1 and EHMT2 are significantly elevated in the PFC	[31]
	Human brain, Mice	H3K4me3	H3K4me3 and its catalytic enzymes are significantly elevated in the PFC in AD	[30]
	Human brain	H4K20me2, H3K4me2, H3K27me3, H3K79me1, H3K79me2, H3K36me2, H4K20me3, H3K27me1, H3K56me1	H4K20me2, H3K4me2, H3K27me3, and H3K79me1 increased, while H3K79me2, H3K36me2, H4K20me3, H3K27me1, and H3K56me1 decreased in AD	[20]
	Mice	H3K4me3	Histone methylation is actively regulated in the hippocampus and facilitates long-term memory formation	[28]
	Mice	H3K4me2, H3K4me3	KMT2B mediates hippocampal H3K4me2 and H3K4me3, which is critical for memory formation	[27]
	Mice	H3K9me2	Increased H3K9me2 levels in the cerebral cortex region and hippocampus region	[86]
	Mice	H3K9me2	NEP is significantly reduced in AD, and hypoxia may downregulate NEP by increasing H3K9me2	[32]
	Mice	H3K4me	A decrease in H3K4 methylation, resulting from KMT2A knockdown, partially recapitulates the pattern previously reported in CK-p25 mice	[29]
	Human brain	H2BK108me, H4R55me	Reduced H2BK108 and H4R55 methylation in the frontal cortex region	[39]
Phosphorylation	Human brain	H4S47p	H4S47p increases in AD	[35]
	Human brain	H2AXS139p (γH2AX)	In the hippocampus region and cerebral cortex region, γH2AX significantly increased	[38]
	Human brain	H3S10p	Activated H3S10p in AD is restricted to the neuronal cytoplasm	[36]
	Human brain	H3 phosphorylation	H3 phosphorylation increases in AD	[37]
Ubiquitination	Human brain	H2BK120ub	H2BK120ub increases in the frontal cortex of AD	[39]
	Human neurons	H2A ubiquitination	Bmi1/Ring1 protein complex maintains the transcriptional inhibition of developmental genes through H2Aub	[41]
	Mice	H2B ubiquitination	A deficiency of H2Bubi in the hippocampus prevents learning-induced increases in H3K4me3, gene transcription, synaptic plasticity, and memory formation	[40]

Histone acetylation, a process that promotes gene expression, is particularly important in regulating gene expression associated with learning and memory [16, 17]. Histone acetylation disorder exists in AD [18–21]. Marzi et al. quantified the genome-wide pattern of H3K27ac and revealed that H3K27ac is closely related to Aβ and tau pathology-related genes [19]. Nativio et al. found that the increase of H3K27ac and H3K9ac is associated with the transcription, chromatin and disease pathway of AD through epigenome analysis. Furthermore, they found that elevated H3K27ac and H3K9ac exacerbates amyloid-β42-driven neurodegeneration in a fly model of AD [20]. Moreover, the expression of H3K122ac and H4K16ac is downregulated in AD [20, 21]. Beyond specific histone sites, the interplay between acetylation regulatory enzymes and AD has also drawn significant attention. These enzymes affect AD neuropathology through different mechanisms, including effects on neuronal synaptic plasticity, Aβ deposition, inflammatory factors, and apoptosis [22–26]. Xu et al. found that the knockdown of 300/CBP reduces H3K27ac, inhibits the expression of genetic programs compensating for increased Aβ load, and leads to increased Aβ secretion [23]. Lin et al. found that the downregulation of Acyl-CoA synthetase short-chain family member 2 (ACSS2) mediates the reduction of ionotropic glutamate receptors through histone acetylation, which aggravates the damage of synaptic plasticity in AD. Conversely, ACSS2 upregulation or acetate supplementation could mitigate these deficits [22]. The modulation of acetylation may emerge as a targeted therapy to prevent or reverse the pathology and disease progression of AD.

Various molecular biology techniques were used to detect changes in methylation levels at different histone sites in the human brain and AD mouse models. Among them, H3K4me3 and H3K9me2 have been extensively studied. Elevated levels of H3K4me3 in the prefrontal cortex (PFC) and hippocampus are associated with the formation of long-term memory and the amelioration of memory deficits [27–30]. Similarly, elevated levels of H3K9me2 were detected in both mouse and human PFC regions [31]. Hypoxia may downregulate NEP (an enzyme responsible for Aβ degradation) by increasing H3K9me2 and decreasing H3 acetylation, which leads to Aβ accumulation, neurodegeneration, and AD [32]. In a study using histone methyltransferase inhibitors to treat AD mice, researchers found that histone hypermethylation can be reversed, leading to the restoration of glutamate receptor expression and excitatory synaptic function in the PFC and hippocampus [31]. Additionally, H3K4me3 and H3K9me2 are also regulated by histone methyltransferase and histone demethylase, which affect the proliferation and differentiation of neurons and the cognitive abilities of AD patients [30, 33, 34].

The phosphorylation levels of histones H3 and H4 increase in the brains of AD patients [35–37]. Ogawa et al. hypothesize that the increased phosphorylation of H3 in AD is confined to the cytoplasm of neurons, indicating a mitochondrial catastrophe that leads to neural dysfunctions and neurogenesis in AD [36]. Researchers have found a significant increase in γH2AX (phosphorylation of serine 139 on H2AX) in astrocytes in hippocampal and cerebral cortex regions in AD [38]. Additionally, ubiquitination of H2BK120 is heightened in the frontal cortex of AD patients [39], while H2B ubiquitination regulates histone crosstalk in learning through non-proteolytic proteome function [40]. Flamier et al. found that the Bmi1/Ring1 complex compresses developmental gene transcription via histone H2A mono-ubiquitination [41].

In summary, classical modifications occurring in different parts of the cerebral cortex and hippocampus affect memory, learning ability, neurodegeneration, and the development of AD through various pathways mediated by different factors. Despite their significance, these modifications cannot fully explain the pathogenesis of AD and facilitate the development of effective treatments, emphasizing the necessity for further research.

## Summary of novel HPTMs processes

In 2007, Zhao’s team first reported two new lysine modifications, propionylation and butyrylation, initiating the discovery of a series of novel HPTMs [42]. Research into the specific regulatory mechanisms and physiopathological effects of these novel HPTMs, as well as their relationship with disease, has since expanded. Novel HPTMs have been reported to be closely associated with multiple biological processes and diseases, such as neurodegenerative diseases [43], renal diseases [44, 45], metabolic diseases [46], cardiovascular diseases [47, 48], cancer [48, 49], and HIV latency [50], thereby becoming a research focus in the epigenetics field.

Intracellular metabolites, which are substrates for ATP production and donors of HPTMs, are key to controlling gene transcription and protein translation [48]. For example, acetylCoA, derived from the glycolipid metabolic pathway, can activate acetylation modifications of histones to enable cells to handle changes in the metabolic environment [51]. Furthermore, some specific acyl-CoAs, such as butyryl-CoA and crotonyl-CoA, as well as *β*-hydroxybutyrylation-CoA, originate from the short-chain fatty acids generated by intestinal microbiota, providing a basis for the exogenous regulation of histone modifications affecting transcription.

Histone acylation modification involves the transfer of acyl-CoA onto histone protein amino acid residues and is tightly and dynamically regulated by multiple enzymes or non-enzymes. These novel HPTM regulatory enzymes substantially overlap with classical acetylases, including acyl-CoA synthetases, acyltransferases, and deacylases, such as ACSS2, p300/CPB, HATs, and HDACs. The association of these regulators with the disease is equally noteworthy [52]. HDACs in mammalian cells are classified into four classes: I (HDAC1, 2, 3, 8), II (HDAC4, 5, 6, 7, 9, 10), III (sirtuins 1–7), and IV (HDAC 11). Several HDACs (HDAC1, 2, 3, 6, 7, 8) and sirtuins (1, 2, 3, 6, 7) have been shown to regulate Kcr levels [53]. These regulators may become therapeutic targets and new diagnostic markers for AD. The deletion of HDAC1 and HDAC2 from mouse microglia ameliorates cognitive deficits and reduces amyloid levels by increasing amyloid phagocytosis in the microglia [54]. HDAC inhibitors have been used to treat neurodegenerative diseases due to their potential neuroprotective mechanisms through upregulating neurotrophic factors, preventing neurotoxic proteins and peptides from accumulating, and downregulating pro-inflammatory cytokines [24]. Additionally, one previous study showed that the serum concentrations of SIRT1, SIRT3, and SIRT6 were inversely related to AD [55]. A receiver operating characteristic analysis demonstrated that these serum proteins exhibit high precision in the diagnosis of AD [55]. As shown in Table 2, a more in-depth exploration of the intricate link between novel HPTMs and AD will be beneficial for identifying additional targeted biomarkers and essential therapeutic targets.

**Table 2:** Summary of novel HPTMs in AD

Novel PTMs	Models	Sites	Gene/target pathways involved	Specific functions	References
Crotonylation	Wild-type and APPswe/PS1dE9 double transgenic mice (an AD mouse model), U251 cells transfected with the siNEAT1v2, siNEAT1(v1 + v2) or negative control siRNA	H3K27	NEAT1, STAT3, CAV2, TGFB2, TGFBR1	It can regulate endocytosis-related gene expression to inhibit the uptake of Aβ in AD	[61]
	N2a/APP695swe cells	Pan-Kcr	–	–	[66]
Lactylation	Brain tissue from AD mouse models, AD patients, BV2 cells	Pan-Kla; H4K18la; H4K5la; H4K8la; H3K18la; H3K23la	Glycolysis/H4K12la/PKM2 positive feedback loop	A glycolysis/H4K12la/PKM2 positive feedback loop that exacerbates microglial activation and dysfunction in AD	[67]
	N2a/APP695swe cells, APP/PS1 Mice	Pan-Kla	–	Catalpol may play a neuroprotective role in AD by modulating lactylation	[68]
β-hydroxybutyrylation	–	–	–	–	–
succinylation	Neurons from embryos were prepared from the cerebral cortices of E15.5 C57BL/6 mice	Pan-Ksucc	KGDHC	KGDHC can serve as a trans-succinylase that mediates succinylation in an *α*-ketoglutarate-dependent manner	[76]
	Human brain tissue samples, transgenic mouse models of AD	Pan-Ksucc	–	Succinylation of APP disrupts its normal proteolytic processing, thereby promoting Aβ accumulation and plaque formation; in addition, succinylation of tau promotes its aggregation into tangles and impairs microtubule assembly	[4]
	Escherichia coli BL21/DE3 cells	Tau succinylation in residue K311	–	K311 succinylation locally perturbs the binding of the tau MBD to tubulin	[80]
	N2a/APP695swe cells	Pan-Ksucc	–	–	66]
Malonylation	N2a/APP695swe cells	Pan-Kmal	–	–	[66]
2-hydroxyisobutyrylation	N2a/APP695swe cells	Pan-Khib	–	–	[66]
	N2a/APP695swe cells, APP/PS1 mice	Pan-Khib	–	–	[68]

## Current advances of novel HPTMs in AD

### Crotonylation

Kcr, first discovered by Zhao’s team in 2011 [10], is a short-chain lysine acylation using crotonyl-CoA as the substrate, and its regulatory factors highly overlap with those of Kac [56]. Histone Kcr is particularly enriched in the transcriptional start sites (TSSs) of mammalian genomes and has a specific *α*, *β* unsaturated carbonyl structure; thus, its effect on transcription is stronger than that of histone Kac [10, 56, 57]. As a popular research topic in recent years, Kcr has been associated with various pathophysiological processes and diseases, including embryonic development [58], neurodevelopment [56], neural differentiation [59], neuron flammation [60], AD [61], neonatal hypoxic-ischemic encephalopathy [62], acute kidney injury [44], HIV [63], and even depression [43]. Recently, Kcr has been shown to modulate the expression of endocytosis-related genes, which modulate the microglia-mediated clearance of Aβ in AD [61]. Stimulating Aβ clearance is considered one of the most promising potential approaches for treating AD. Microglia play a central role in the progression of AD because of their ability to remove soluble Aβ protofibrils and protofibrillar Aβ through both endocytosis and autophagy [61, 64, 65]. Wang et al. found that nuclear paraspeckle assembly transcript 1 (NEAT1) is dysregulated in AD [61]. Mechanistically, through acylation modification histology, ChIP-seq, and other approaches, NEAT1 has been confirmed to change the acylase activity of P300 by binding to P300 and changing H3K27ac and H3K27cr, which are situated close to the TSSs of a number of gene promoters, as well as by downregulating endocytosis-related genes to inhibit Aβ uptake in AD [61]. Another study demonstrated that crocetin, the active ingredient of saffron, can exhibit neuroprotective effects in AD via the downregulation of Kcr [66]. Regrettably, due to the limited research on Kcr, there is still a lack of clarity regarding its exact regulatory role in the pathogenesis of AD, which needs to be explored in further clinical and basic studies.

### Lactacylation

Lactate is a metabolite of cellular anaerobic glycolysis that is commonly considered to be a metabolic waste product with temporary energy-supplying properties. However, lactate has recently received increasing attention due to its other biological roles, including substance-energy metabolism, neurotransmitter transmission, and neurovascular coupling. In particular, the discovery of Kla has greatly broadened the biological significance of lactic acid, which exerts nonmetabolic functions; this has provided insight into the pathogenesis of several diseases. Kla, as a novel HPTM, is dynamically regulated by cellular metabolism-produced lactate, and it can directly influence the processes of gene replication, transcription, and translation, thus affecting the biological effects of cells [11]. Pan et al. discovered that Kla is a critical player in AD pathogenesis [67]. Specifically, they found that H4K12la is highly enriched in the promoter regions of microglia glycolysis-related genes and that it activates the transcription of these genes to promote microglial glycolytic activity, leading to positive feedback regulation and the intensification of microglia dysfunction in AD [67]. This study shows that metabolic disorders have an important role in neuroinflammation and the initial phases of AD, indicating novel directions for early intervention in AD [67]. Furthermore, a different study suggested that catalpol likely plays a neuroprotective role in AD by modulating Kla [68]. Consequently, Kla may emerge as a potential target for future AD therapy; however, several questions remain to be investigated, including the exact regulators and regulatory sites of lactoylation, as well as how lactoylation dynamically changes and collaborates with other types of acylation.

### β-hydroxybutyrylation

β-hydroxybutyrate (BHB) is an endogenous ketone produced by metabolism. The level of BHB has been found to be decreased in patients with AD [69].

BHB can improve cognitive function and alleviate lesions in patients with AD via various mechanisms, such as modulating signaling molecules, adjusting intestinal flora, impacting Aβ and tau protein formation, enhancing mitochondrial metabolism, suppressing inflammation and lipid metabolism, and enhancing histone Kac [70]. The neuroprotective effect of BHB shows its potential as a therapeutic drug for improving cognitive function in AD patients. At present, donepezil, an acetylcholinesterase inhibitor, is used to treat AD and increases the plasma level of BHB, improving cognitive function [69]. A randomized controlled trial also confirmed that ketogenic drinks containing BHB could improve the cognitive outcomes of patients with cognitive impairment [71]. BHB is a high-energy donor of histone Kbhb, and it can bind to free CoA to form β-hydroxybutanoyl CoA [72]. Kbhb is a recently identified HPTM that is closely related to ketone metabolism. BHB is an important substrate of Kbhb, and evidence for its association with AD has been increasingly demonstrated. BHB can alleviate the pathological changes of AD and enhance cognitive function via various mechanisms; however, the therapeutic mechanism of Kbhb in AD has not been confirmed, and more research into the specific epigenetic mechanisms is required.

### Succinylation

Ksucc is generated by the reaction between succinyl-CoA, a dicarboxylic acid compound metabolized from amino acid residues that is directly coupled to the tricarboxylic acid (TCA) cycle, as well as amino acid residues [13, 73]. Ksucc increases the structural moiety and charge changes to a greater extent than Kac or Kme. Accordingly, the more dramatic structural changes caused by Ksucc might induce greater alterations in protein structure and function [13]. Ksucc has a wide cellular distribution, particularly in mitochondria, and is known to regulate protease activity and gene expression [74]. Additionally, it is involved in various life processes, including glucose, amino acid, and fatty acid metabolism, as well as ketone synthesis and the scavenging of reactive oxygen species [73, 75]. The α-ketoglutarate dehydrogenase complex (KGDHC) is markedly reduced in the mitochondria of patients with AD [76]. The extensive decrease in post-translational Ksucc in the regional brain is caused by the suppression of KGDHC activity [4, 77]. Some studies have shown that KGDHC is located in the nucleus and combines with lysine acetyltransferase 2A in the promoter region of the gene to participate in the Ksucc modification of histone H3 [78]. However, the exact targets of H3 are unknown. Additionally, SIRT5, a desuccinylase, increases during AD progression [79]. It was recently confirmed that the combined effect of changes in these regulators leads to the downregulation of Ksucc in AD. A proteomic profiling study identified decreased Ksucc modification in multiple mitochondrial proteins and increased Ksucc modification of a small number of cytosolic proteins in AD [4]. Remarkably, the most significant increases took place in key APP and microtubule-associated tau sites [4]. Furthermore, studies have shown that Ksucc modification of APP contributes to Aβ accumulation and plaque formation by disrupting normal protein hydrolysis processing, while Ksucc modification of tau facilitated its aggregation into tangles and impaired microtubule assembly [4, 80]. Studies on Ksucc have primarily focused on its association with mitochondrial metabolic pathways, and the specific targets and regulators of histone Ksucc are largely unknown. Therefore, it is essential to investigate the specific roles, as well as the precise regulatory mechanisms, of histone Ksucc in AD models to meet drug development needs. In summary, Ksucc may become a potential diagnostic indicator and therapeutic target in AD.

### Malonylation and 2-hydroxyisobutyrylation

Kmal and Khib were discovered by Zhao’s team in 2011 and 2014, respectively [14, 15]. Increasing research has since been conducted on these two novel acylation modifications. Du et al. used Neuro-2a cells, which can produce a mass of Aβ and consistently express N2a/APP695swe (the APP695 gene with a Swedish family mutation) to explore the role of catalpol and crocin in neuroprotection against endogenous Aβ-induced neurotoxicity [66, 68]. The crocin experiment indicated that crocin inhibited apoptosis of endogenous Aβ neurons through the mitochondrial pathway [66]. Another study showed that catalpol ameliorated neurological damage and alleviated cognitive impairment in N2a/APP695swe cells and APP/PS1 mice by modulating HPTMs, reducing apoptosis, alleviating Aβ production, and attenuating mitochondrial damage [68]. Importantly, both studies revealed changes in novel HPTMs: in crocin-treated N2a/APP695swe cells, Ksucc, Kcr, Khib, and Kmal were significantly reduced [66], while slight alterations in Kla and Khib levels were found in catalpol-treated N2a/APP695swe cells [68]. The regulation of novel HPTMs may represent a new mechanism for drug intervention to improve AD. The aforementioned findings significantly contribute to our understanding of potential novel treatment targets in AD, and provide insights for diagnostic and treatment methods aimed at HPTMs. The findings are particularly promising in the context of the challenges and prospects associated with research focused on identifying new therapeutic targets.

## Conclusion and perspectives

**Fig. 1:**
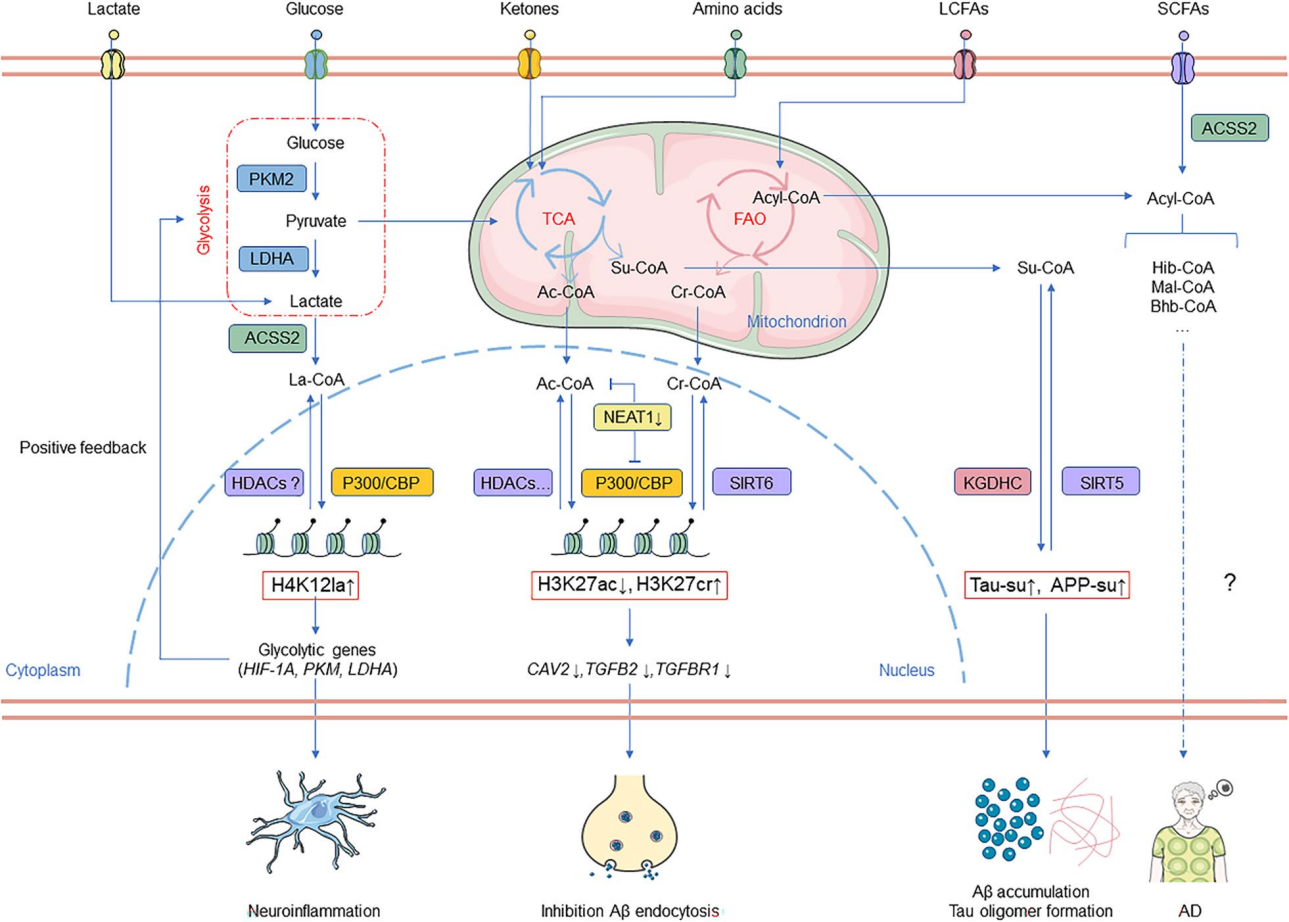
Function and mechanism of various novel HPTMs in AD. Fatty acids, amino acids, glucose, ketones, and lactic acid generate various acyl-CoAs, which are substrates for various novel HPTMs. Lactic acid-induced H4K12la activates the transcription of glycolysis-related genes (including HIF-1A, PKM, and LDHA), thus exacerbating neuroinflammation in AD. NEAT1 changes the acylase activity of P300 by binding to P300 and increasing H3K27ac/H3K27cr, which downregulates endocytosis-related genes (CAV2, TGFB2, and TGFBR) and inhibits the uptake of Aβ in AD. KGDHC and su-CoA in mitochondria efflux into the cytoplasm, resulting in the upregulation of APP and tau succinylation, promoting Aβ accumulation, plaque formation, and tau aggregation into tangles. Kbhb, Kmal and Khib have been shown to be closely associated with AD; however, their exact mechanisms of action remain unclear. AD Alzheimer’s diseaseβ; LCFA, Long-chain fatty acid; SCFA, Short-chain fatty acid; TAC, Tricarboxylic acid cycle; FAO, Fatty acid oxidation; HIF-1A, Hypoxia-inducible factor-1A; PKM, Pyruvate kinase M; LDHA, Lactate dehydrogenase A; NEAT1, Nuclear paraspeckle assembly transcript 1; ACSS2, Acetyl-CoA synthetase; P300, An acetyltransferase; CBP CREB-binding protein; HDACs Histone deacetylases; SIRT, Sirtuin; APP, Amyloid Precursor Protein; CAV2, Caveolin 2; TGFB2, Transforming growth factor-beta 2; TGFBR, TGF-beta receptor; KGDHC, α-ketoglutarate dehydrogenase complex; su-CoA, Succinyl-CoA; ATP, Adenosine triphosphate; Kbhb, β-hydroxybutyrylation; Ksucc, succinylation; Khib, 2-hydroxyisobutyrylation; Kmal Malonylation

Recent findings indicate that epigenetics and HPTMs, especially acetylation and novel acylation modifications, play a crucial role in AD pathogenesis, especially in Aβ accumulation and plaque formation. As shown in Fig. 1, we review the research on various HPTMs in AD, with a special focus on various novel modifications, to provide a fresh perspective for future exploration into the pathophysiology of AD and the development of new drugs and diagnostic methods.

Meanwhile, due to the overlap of multiple modified regulatory enzyme systems, it is particularly important to identify specific regulatory factors to prevent adverse reactions to new drugs. Acylation modification changes are dynamic and reversible. Various acylation modifications are regulated by various regulatory factors and are dynamically regulated in response to metabolic levels, such as BHB. Therefore, exogenous supplementation of modified precursor substances or the regulation of modified enzyme activity may represent a new therapeutic approach for AD.

It is worth noting that research on novel HPTMs is still relatively limited compared to that on classical HPTMs, such as acetylation and methylation. Many of these studies are limited to observing changes in the levels of HPTMs and related factors, without identifying specific action sites. In addition, some new acylation modifications (such as glutarylation and benzoylation) have not been thoroughly studied in the context of AD. The scarcity of research may be attributed to the high cost of specific antibodies and the absence of specific inhibitors for novel acylation. With the rapid development of new site-specific antibodies, acylation modification detection methods and global maps will greatly expand in the future. Although there is currently no cure for AD, drugs developed based on epigenetics and novel HPTMs are likely to effectively improve disease symptoms and prognoses in the future. Further in-depth exploration of the biological effects and regulation of novel HPTMs will broaden our perspective on the treatment of AD.

## Data Availability

Data will be made available upon request.

## Abbreviations

AD: Alzheimer’s disease
HPTMs: Histone post-translational modifications
Aβ: Misfolded amyloid beta
FDA: The US food and drug administration
Kcr: Crotonylation
Kla: Lactoylation
Kbhb: *β*-Hydroxybutyrylation
Ksucc: Succinylation
Khib: 2-Hydroxyisobutyrylation
Kmal: Malonylation
Kac: Acetylation
Kme: Methylation
P: Phosphorylation
Kub: Ubiquitination
ACSS2: Acetyl CoA transferase
PFC: Prefrontal cortex
NEP: An enzyme responsible for Aβ degradation
γH2AX: Phosphorylation of serine 139 on H2AX
TSSs: Transcriptional start sites
NEAT1: Nuclear paraspeckle assembly transcript 1
BHB: *β*-Hydroxybutyrate
TCA: Tricarboxylic acid
KGDHC: *α*-Ketoglutarate dehydrogenase complex
N2a/APP695swe: The APP695 gene with a Swedish family mutation
